# Editorial: Zinc in health and disease management

**DOI:** 10.3389/fnut.2023.1242840

**Published:** 2023-07-10

**Authors:** Héctor González-Iglesias, Imre Lengyel, Benjamin Rolles

**Affiliations:** ^1^Instituto de Productos Lácteos de Asturias, Consejo Superior de Investigaciones Científicas (IPLA-CSIC), Villaviciosa, Spain; ^2^Wellcome-Wolfson Institute for Experimental Medicine, School of Medicine, Dentistry and Biomedical Science, Queen's University Belfast, Belfast, United Kingdom; ^3^Department of Hematology, Oncology, Hemostaseology and Stem Cell Transplantation, Medical Faculty, RWTH Aachen University, Aachen, Germany; ^4^Center for Integrated Oncology Aachen Bonn Cologne Duesseldorf (CIO ABCD), Bonn, Germany; ^5^Brigham and Women's Hospital and Harvard Medical School, Boston, MA, United States

**Keywords:** clinical nutrition, dietary zinc, zinc supplementation, aging, cancer, zinc homeostasis, X-ray fluorescence, pregnancy

Zinc is an essential trace element with various functions in all living organisms. Its bioavailability is tightly controlled by a combination of nutritional availability, binding to proteins, location in cellular organelles, and the presence of zinc transporters. In humans, changes in zinc homeostasis, such as zinc deficiency, have varied impacts on individuals. Zinc affects growth, fertility, immune cell functioning, and disease progression. Zinc homeostasis is compromised in aging, whereby abnormal metabolism contributes to sub-clinical changes and cellular dysfunction that may underlie the early onset of (neuro-)degenerative diseases. Therefore, a better understanding of the normal function of this trace element in health and diseases is highly desired.

Our Research Topic brings together eight research articles presenting the most recent developments in zinc nutrition. The collection provides new data on zinc and its potential impact on telomere length, the need for novel biomarkers of zinc status, the usefulness of X-ray fluorescence to detect changes in hair zinc concentrations, the effects of dietary zinc during pregnancy, its role during impaired metabolism in retinal cells and its relationship in the context of ovarian as well as skin cancer.

Telomere length is considered a biomarker of aging that dietary and environmental factors can influence. Shi et al. studied the relationship between zinc intake and its potential impact on telomere length among middle-aged and older individuals, using the dataset generated as part of the National Health and Nutrition Examination Survey (NHANES). In this cross-sectional study carried out on 3,793 participants older than 45 years, elevated dietary zinc intake was positively correlated with longer telomere length, especially in women, those with low energy intake and obese individuals. It appears that dietary zinc intake may contribute to changes in telomere length and protect toward telomere attrition.

Frederickson et al. determined the potential of using hair samples to detect changes in zinc concentration in response to the consumption of zinc-biofortified wheat flour. The BiZiFED2 effectiveness trial was set up to combat zinc deficiency in Pakistani girls (10–16 years old). The participants received flour from control or zinc-biofortified wheat for 6 months. Zinc and sulfur content were determined in hair samples of all participants using an inexpensive and portable X-ray fluorescence (XFR) detector. The work demonstrated that a modest increase in dietary zinc over 6 months resulted in a detectable increase in sulfur and zinc, offering a sensitive and non-invasive analytical methodology to monitor changes in response to dietary zinc interventions.

The zinc content of breast milk is critical for the normal development of an infant. A clinical trial conducted by Han et al. investigated the effects of supplements containing vitamins, minerals, zinc and probiotics on zinc concentrations in breast milk. They found that breast milk of women receiving supplements had higher zinc concentrations at 3 and 6 months of lactation but levels decreased by 12 months. One of the interesting findings was that zinc supplementation from as early as preconception affected the zinc concentrations during lactation. Therefore, early supplementation can ensure adequate maternal zinc status and adequate zinc transfer to the infants. A second, this time case-control study from Palestine by El Bilbeisi et al. assessed dietary and serum zinc levels during the third trimester of 160 pregnant women with and without pregnancy-induced hypertension (PIH). It is rare to see publications from this region. The authors found that the main risk factors of PIH included a familiar history of hypertension, primiparous, previous caesarian section, preeclampsia, oedema and, of course, low maternal dietary zinc intake, highlighting the importance of appropriate zinc status during, and probably, before pregnancy.

Zinc status is also important for the older generations. Previous clinical studies conducted by the Age-Related Eye Disease Study (AREDS) showed that dietary supplementation of zinc and vitamins slows the progression of age-related macular degeneration (AMD), one of the major causes of irreversible vision loss in people >65 years. One of the cellular targets in AMD is the retinal pigment epithelium (RPE). Álvarez-Barrios et al. used a human fetal RPE cell culture model that recapitulates features of early AMD. The authors specifically studied metallo-transcriptomics and found that altered zinc homeostasis changed the expression of cytosolic zinc-binding proteins, zinc transporters, and metalloproteins, supporting the growing evidence for the direct role of zinc dyshomeostasis in AMD.

Considering that dietary intake of trace elements could be relevant for the prevention or prognosis of cancer, Yin et al. explored the relationship between pre-diagnostic dietary copper and zinc and the copper-to-zinc (Cu/Zn) ratio in 701 women diagnosed with ovarian cancer (OC). Higher dietary Cu intake and altered Cu/Zn ratio were related to patients diagnosed as non-serous OC, suggesting that supplementation with trace elements can provide treatment options for longer survival time. Tang et al. carried out a logistic regression analysis on data from NHANES to evaluate serum copper and zinc levels in patients with cutaneous melanoma and non-melanoma skin cancers (NMSC). Not significant relationships were found between serum zinc and the Cu/Zn. However, higher serum copper levels might be associated with a decreased incidence of NMSC. These early findings on different cancers suggest that there is a need for larger, prospective cohort studies to confirm these associations.

Finally, there is a need for adequate, sensitive and specific biomarker for zinc status. The review article by Knez and Boy summarizes the current knowledge and recommendations focusing on how to evaluate zinc status in humans. Currently, plasma zinc level can indicate severe zinc deficiency but it is insensitive to detect mild/moderate and early-stage deficiency. The authors discussed the potential to use the gene expression profiles of fatty acid desaturases 1 and 2 (FDAS1/FADS2), the linoleic acid/dihomo-γ-linolenic acid ratio, thymulin levels, changes in the gut microbiome and the presence of different zinc transporters to accurately determine zinc status.

This Research Topic demonstrates the continued interest and the importance of zinc as an essential trace element. With the development of more sensitive and specific biomarkers for zinc status and the evolving routes to increase zinc nutrition we should be able to eradicate the zinc deficiency associated comorbidities faster.

## Author contributions

HG-I, IL, and BR wrote, revised this article, and co-edited the Research Topic. All authors made a substantial, direct, and intellectual contribution to this work and approved the final version for publication.

